# Design and Implementation of Website Information Disclosure Assessment System

**DOI:** 10.1371/journal.pone.0117180

**Published:** 2015-03-13

**Authors:** Ying-Chiang Cho, Jen-Yi Pan

**Affiliations:** Department of Electrical Engineering, National Chung Cheng University, 168 University Road, Chia-Yi 62102, Taiwan, R.O.C; University of Catania, ITALY

## Abstract

Internet application technologies, such as cloud computing and cloud storage, have increasingly changed people’s lives. Websites contain vast amounts of personal privacy information. In order to protect this information, network security technologies, such as database protection and data encryption, attract many researchers. The most serious problems concerning web vulnerability are e-mail address and network database leakages. These leakages have many causes. For example, malicious users can steal database contents, taking advantage of mistakes made by programmers and administrators. In order to mitigate this type of abuse, a website information disclosure assessment system is proposed in this study. This system utilizes a series of technologies, such as web crawler algorithms, SQL injection attack detection, and web vulnerability mining, to assess a website’s information disclosure. Thirty websites, randomly sampled from the top 50 world colleges, were used to collect leakage information. This testing showed the importance of increasing the security and privacy of website information for academic websites.

## Introduction

### 1. Research motivation

As technology and the Internet grow more pervasive, web vulnerabilities increasingly threaten website information security [1, 2]. Many malware and malicious technologies, such as spam and advanced persistent threats (APTs), have been designed during the past 20 years [3, 4]. Attackers usually focus on two web vulnerabilities: e-mail address leakage and website database leakage. The former is typically caused by web programmers’ negligence to filter the most significant symbol, @, in e-mail addresses. This symbol is easily detected by disclosure mining systems. Website database leakage can be generated using the error settings of “robots.txt” files, which are used by most applications, and can prevent web crawler programs from accessing web pages [5, 6]. When crawlers attack, they first access a certain page and test whether a “robots.txt” file exists. Though this file is of great importance for web information security, it is not always enforced due to various programming and administration mistakes. To avoid being bypassed by malicious applications, more attention should be paid to password settings and improving the program writing techniques [7–10].

A website information disclosure assessment system, equipped with a black-box testing mechanism, is proposed to solve these two chief problems [11–13]. There are three main modules in this system: the dynamic scanning, static mining, and manual operating modules. These three modules serve six functions: data syntax analysis, hidden page exploration, multi-domain searching on one Internet Protocol (IP), specific file searching, search engine assistance, and website vulnerability updating.

**Fig 1:**
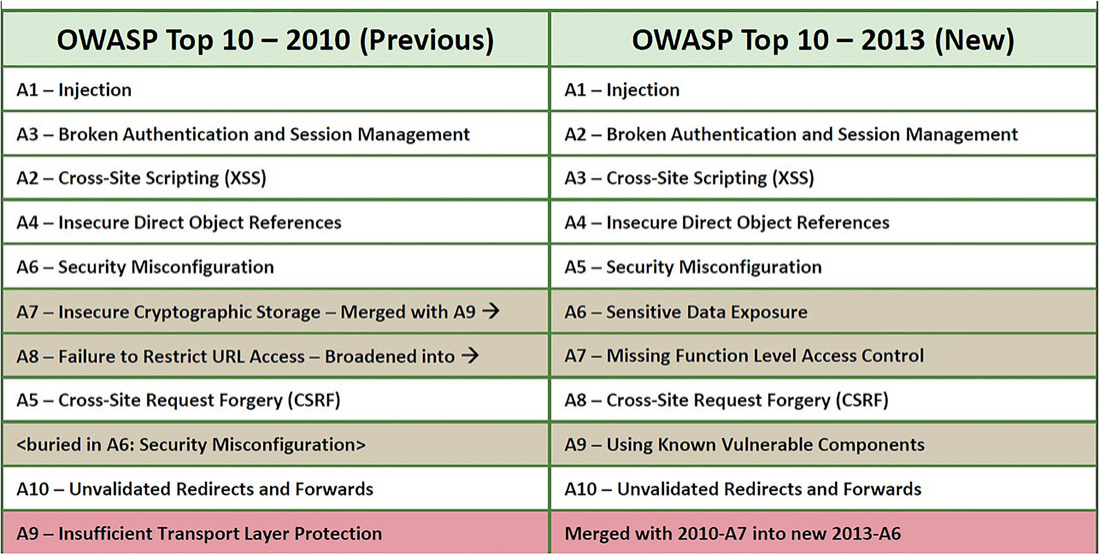
Top 10 network security threats.

According to statistics from the Open Web Application Security Project (OWASP) 2013, injection is the biggest threat to security vulnerabilities. This is based on web applications from 2010 until now and can be seen in Fig. 1 [14].

System security vulnerabilities, also known as system vulnerability, are defined in RFC2828 [15] to be: “A flaw or weakness in a system’s design implementation or operation and management that could be exploited to violate the system’s security policy.”

This paper is organized as follows. Section II introduces the core system and techniques. Section III clarifies the system implementation. Section IV presents experimental results and analyses. Section V compares different applications. Section VI discusses response strategies, and Section VII offers a conclusion.

### 2. Introduction of the core system and techniques

#### 2.1 Dynamic analysis

A dynamic analysis tool directly finds problems in an operating web page, browses the page by simulating the harmless behaviors of users, cooperates with automation tools to analyze the web page content, generates requests with different parameters according to the analysis results, and then analyzes response results in order to discover known or unknown vulnerabilities [16–19]. These vulnerabilities are real security problems obtained by simulating the user’s behavior, which is unlike the misreported problems given by the original code detection. Based on static and dynamic testing technologies, an increasing number of special detection methods appeared, such as black-box testing [20], fuzz testing [21], and penetration testing [22]. Black-box testing determines vulnerability by analyzing the testing responses from an application’s numerical input. On the other hand, white-box testing, which only analyzes source codes, is relatively ineffective for online applications between the web server, application server, and database server. Therefore, while testing web applications, black-box testing is more commonly used to test and observe the response. Fuzzing, based on injection defects, is an automatic software testing technology, which inputs a large number of effective data (semi-values) into an application and tests the program for irregularities, thereby finding the application’s security vulnerabilities. False positives are uncommon with fuzzing because its dynamic execution has a high degree of automation, unlike a static analysis, which requires a substantial amount of human involvement during the reverse engineering process [47]. As a result, fuzzing technology is a fairly effective and low-cost method. This is the reason many companies and organizations use it to improve the quality of their software, vulnerability analysts use it to find bugs, and hackers use it to attack. Penetration testing evaluates the security of a computer system or network using simulated attacks [48]. This approach analyzes all possible weaknesses of the system. The testing results are valuable and compelling. However, this technology not only has its weakness but also can be exploited and used to attack. Honest testing results create a communication bridge between developers and the information security, which allows setting of achievable goals and consequently prompts developers to fix problems [49].

#### 2.2 Injection technology

Various injection technologies, such as SQL and Shell injection, are increasingly attracting attention. SQL injection [23, 24] is a code injection technique used to attack data-driven applications, in which malicious SQL statements are inserted into an entry field for execution. For example, the executed statements may dump database contents to the attacker. In order to be effective, an SQL injection must exploit a security vulnerability in an application’s software. Using the action of a regular SQL query, SQL injection injects attack program instructions into the query commands, penetrates the firewall, bypasses the identity authentication mechanism, and obtains control of the database in order to view and modify the data. In current web system development environments, such as ASP, PHP and JSP, SQL injection is popularly used to generate logic errors that destroy different kinds of databases. Shell injection, also known as command injection, is generally considered one of the most dangerous vulnerabilities because it can be used to gain complete control over a target server. Although server and OS hardening limit the impact and make it more difficult for an attacker to gain privileges, a significant risk still exists. Oftentimes, web applications need to take advantage of their underlying programs or applications in order to complete some function. This may be as simple as sending an e-mail using the Unix sendmail program or as complicated as running custom Perl and C++ programs. From the development point of view, this is an excellent way to reduce the development time of an application. However, if data is passed to these programs via a user interface, an attacker may be able to inject shell commands into these backend programs, potentially leading to compromise.

#### 2.3 Web crawler

A web crawler is an Internet bot that systematically browses the World Wide Web, typically for web indexing. A web crawler may also be called a web spider [25], an ant, an automatic indexer [26], or a web scutter. Web search engines and some other sites use web crawling, or spidering, software to update their own web content or the indices of another site’s web content. Web crawlers copy all the pages they visit for later processing using a search engine that indexes the downloaded pages in order to allow users to search them much more quickly. Crawlers validate hyperlinks and HTML code. They are also used for web scraping [27].

A web crawler starts with a list of URLs, called the seeds. As the crawler visits these URLs, it identifies all the hyperlinks in the page and adds them to the list of URLs to visit, called the crawl frontier. URLs from the frontier are recursively visited according to a set of policies. If the crawler is archiving websites, it copies and saves the information as it goes. Such archives are usually stored so that they can be viewed, read, and navigated as though they were on the live Web but are actually preserved as “snapshots” [28].

#### 2.4 Targeted threats explained: advanced persistent threats and e-mail address leakage

Advanced persistent threats (APTs) [3, 4] are multiple attacks against a specific agency. The main purpose of these attacks is to penetrate the network of a target agency and steal confidential information. Attackers use malicious tools in order to establish a remote-control architecture, similar to botnet, and momentarily steal intelligence. APTs may include intelligence-gathering technology and personnel that can cause an attack to last for a short period. For example, while stealing trade secrets a few months may be spent gathering security protocols, application weaknesses, and file locations. After the intelligence collection is complete, the formal attack will not necessarily last long.

Spear phishing may be defined as “a phishing aiming at some individual or group in a specific agency,” which is similar to fishing with a harpoon [29, 30]. This attack uses information related to the target to adjust contents, making itself appear more specific, or “personalized” for the victims. For example, spear phishing e-mails may use the victim’s name, position, or title, unlike normal phishing, which commonly uses generic names. APT attacks often use spear phishing techniques because victims in higher positions are more tempted to open these e-mails [31, 32]. These targets likely have some knowledge about the company’s information security principles, so they are less likely to open general phishing e-mails or have no time to read messages that appear to be spam. Spear phishing significantly increases the odds of an e-mail being read by the target, which increases the likelihood of penetrating target networks. In many cases, spear phishing e-mails use normal-seeming attachment files because sharing files via e-mail is common in many large enterprises and government agencies. Therefore, these institutions are often the target of APTs.

**Fig 2:**
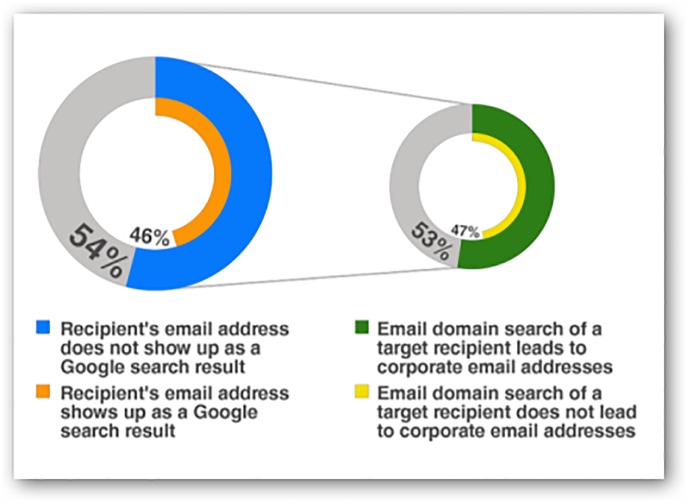
Website search plays an important role in e-mail address selection [33].

The reconnaissance gathered before the penetration occurs mainly focuses on the target agency’s people. In this stage, the hacker acquires personnel information, such as names, titles, and e-mail addresses, from underground markets or attack funders. This information is also conveniently found on the Internet. Attackers collect relevant information needed for their social engineering technique from social networking sites, enterprises, institutions, and academic publication websites [34]. This reconnaissance allows an attacker to find the key personnel of target institutions. These people usually are powerful, have important files, or have permission to access the desired data. Once the key personnel are found, criminals determine their e-mail addresses, which will be used in the spear phishing attacks [33]. Therefore, the ability to obtain a target’s e-mail address using multiple methods and distinguishing the e-mail owner’s property from different areas, like the website, becomes very important. This is shown in Fig. 2.

## Methods

### 3. System implementation

Most modern site security detecting tools only prompt that there are risks in certain parts of a website, but they do not actually attack the target website. Therefore, we hope to understand SQL injection attacks and determine the possible damage by implementing a set of tools and attacking target websites. To achieve these goals, we designed an SQL injection penetration system to test the personal privacy information revealed by target websites. This system utilizes black-box testing, penetration testing, and other technologies. It combines the spirit of web crawlers and the concept of application search engines with vulnerability detection. This system detects whether websites have SQL injection, vulnerability, or an e-mail address leakage [45, 46].

**Fig 3:**
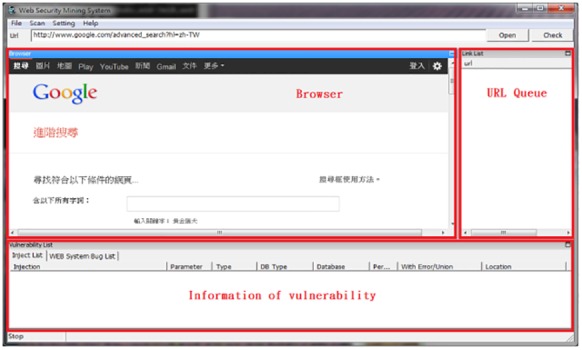
WIDAS interface.

This study used the website information disclosure assessment system (WIDAS), shown in Fig. 3. It was developed according to the previously mentioned algorithms, using JAVA SE7 with more than 11,000 rows of coding. It can be installed normally in the Java Runtime Environment (JRE) on WinXP, Vista, Win7, or Win8. WIDAS can perform injections that penetrate databases, such as MS-SQL, MySQL, Oracle, PostgreSQL, SQLite, and Access, as well as web languages, such as ASP, ASPX, PHP, JSP, and CGI.

**Fig 4:**
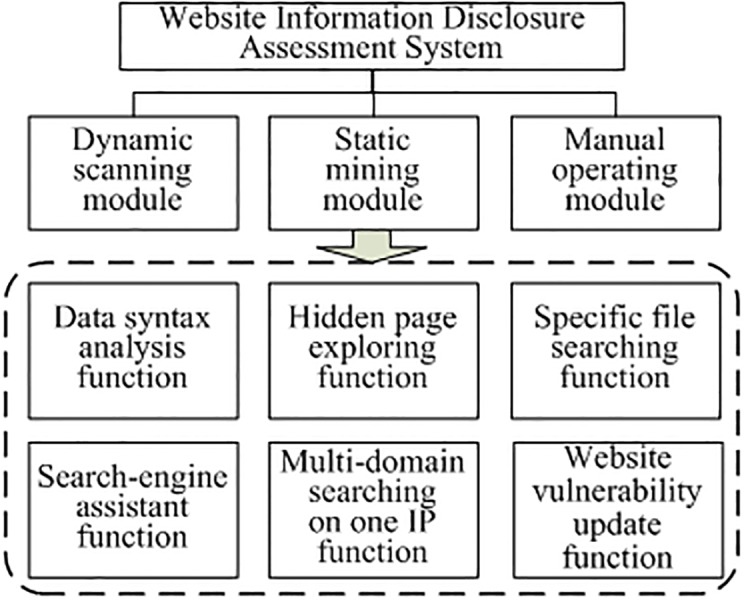
WIDAS framework.

WIDAS contains three modules and six functions [45, 46], as is shown in Fig. 4. The modules are dynamic scanning, static mining, and manual operating modules. The dynamic scanning module detects multiple websites using a keyword query on different search engines in the market and the leakage detection function of the proposed system. The static mining module makes a deep detection on a single site, such as e-mail leakage, the presence of robots.txt files, an SQL injection, file downloading URLs, or broken links. The crawler, injection, and scheduler are the core concerns of this study.

#### 3.1 Crawler

The crawler module mainly analyzes web page content and filters out useful information [34–37]. This module is divided into three components: crawler, crawler queue, and visited table. The crawler crawls web pages and filters necessary information, which contains links and e-mail addresses. It is designed as a multithreaded processing program, so it can quickly crawl entire sites. The crawler queue stores the links filtered from the crawler, and the visited table records the links that have been crawling.

At present, many social attacks and APT attacks are based on e-mail information. Therefore, fixed detection focuses on e-mail filtering, and the following example also focuses on links and e-mails when filtering for details.

(1) Link selection:

After downloading a web page, the crawler filters web content information using regular expressions, i.e., “href=\"(http://){1}([^(\")]*)\"”. If this is in the fixed scanning function, then it compares the filtered-out links with the initial link in order to verify that they belong to the same web domain.

def GetAllUrl(url, html):

    
urllist = []

    
reg = re.compile(‘href = \“(http://){1}([^(\“)]*)\”’)

    
urlarray = reg.findall(html)

    
for one in urlarray:

    
…

domain = GetDomain(newurl)

    
…

    
return urllist

When the crawler stores links collected in the crawler queue, the scheduler filters them during their first time through, comparing them with the dictionary library and giving high weight to links having substantial relevance. The dictionary library information was gathered from the SQL injection cases recorded by the Exploit Database [51] and WooYun [52].

asp?id =

cat.asp?cat =

productlist.asp?catalogid =

….

index.cfm?pageid =

Category.cfm?c =

productlist.cfm?catalogid =

In addition to the comparison made with the dictionary library, the scheduler checks the link structure because SQL injection commonly exists in dynamic links. The filter for the scheduler is “?”.

and 8 = 9 and 8 = 8

and user = 0 and ′8′ = ′8′

and 8 = 9 and ′8′ = ′8

%’ and ‘%‘ = ‘

and 1 = 1

def GetMaybeInjectUrl(url, html):

    
urllist = []

    
reg = re.compile(‘href = \“(http://){1}([^(\“)]*)\”’)

    
urlarray = reg.findall(html)

    
for one in urlarray:

    
…

    
if url2.find(‘ = ’) = = -1 or url2.find(‘?’) = = -1:

       
…

       
return urllist

Common websites are excluded and skipped by the crawler, which can be seen below.

commonURL = [‘baidu.com’, ‘google.’, ‘yahoo.com’, ‘msn.com’, ‘live.com’, ‘bing.’, ‘microsoft.com’, ‘joinsmsn.com’, ‘microsofttranslator.com’, ‘googleusercontent.com’, ‘youtube.com’, ‘blogger.com’]

(2) E-mail filtering:

The crawler filters out e-mails during the web content analysis using the regular expression: “([\w-]+(?:\.[\w-]+)*@[\w-]+(?:\.[a-zA-Z-]+)+)”. Formal expressions of e-mail filtering are not limited to the “@” condition but also need to take other factors into account.

def Gete-mail(content):

    
mails = []

    
re_mail = re.compile(r"([\w-]+(?:\.[\w-]+)*@[\w-]+(?:\.[a-zA-Z-]+)+)")

       
for m in ms:

mails.append(m)

    
return mails

#### 3.2 Injection

The injection module detects an SQL injection. If the website programming system neglects to check the SQL commands in the entered values, harmful instructions may be mistakenly assumed to be the normal SQL commands that will cause unexpected feedback data from database. This abnormal feedback information can be obtained by illegal users and may lead to serious information security issues, such as data leaks, site structure detection, system administrator account changes, malicious web page links, and malicious cross-site script insertion [38, 39].

The injection module has three components: injection, injection queue, and injected table. Injection mainly executes the threat detection and penetration testing of the SQL injection. The injection queue stores the links to be detected, and the injected table records the tested links.

This module first determines the site’s design quality by searching for injectable links. It then joins the grammar dictionary library information in the URL and uses the feedback information to automatically determine whether continued digging would be valuable. When it is valuable to keep digging, the next step is to detect the website’s database type using the checking functions defined by different databases. MS SQL and MySQL, for example, use “len ()” to calculate length, while Oracle uses “length ()”. In other words, when “len (‘s’) = 1” is used to test if a website message can be properly given, the target site’s database may be MS SQL or MySQL. Otherwise, Oracle or another database type must be used. In addition, other internal functions can also distinguish database types. This study checks for MS SQL, MySQL, Access, Oracle, SQLite, and PostgreSQL database types.

After obtaining the database type, the table speculation and field detection must be created in different ways. Specific dictionaries are needed in Access, while specific SQL instructions can be used for query tables and fields in MS SQL and some in MySQL.

**Fig 5:**
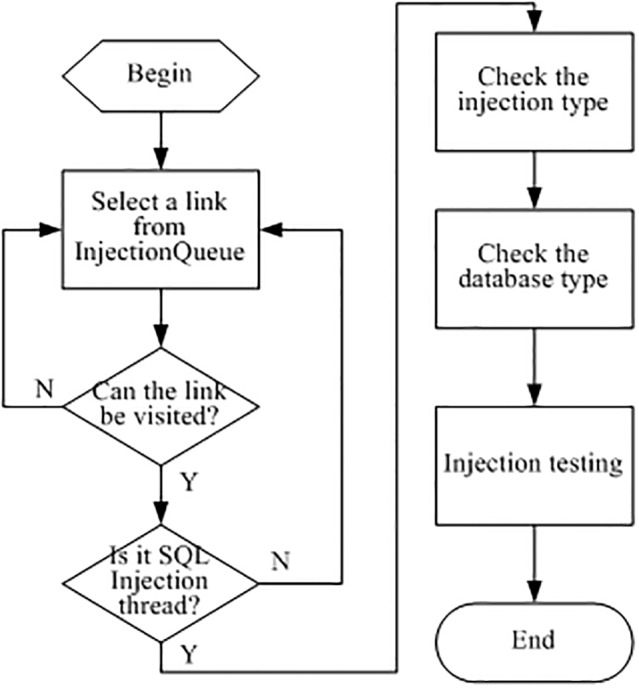
Injection check flow.

Fig. 5 shows the system operation process after the injection point was determined. In order to decipher whether links are injectable, three detection types can be used: integer, string, and searching type injections. Thus, the practical injection detection needs to perform the following check:

def CheckIsInject(self):

        
# check whether it is an injection point.

        
nRet = CheckKey(self.conf,“”,‘int’)

        
if nRet = = False:

            
nRet = CheckKey(self.conf,“”,’str’)

            
if nRet = = True:

                
nRet = CheckKey(self.conf,“and 8 = 9”,’str’)

                
if not nRet:

                    
self.conf.InjectType = u’str’

            
else:

                
nRet = CheckKey(self.conf,“”,’search’)

                
if nRet = = True:

                   
nRet = CheckKey(self.conf,“and 8 = 9”,’search’)

                    
if not nRet:

                        
self.conf.InjectType = u’search’

                
else:

…

        
else:

            
nRet = CheckKey(self.conf,“and 8 = 9”,‘int’)

…

                
self.conf.InjectType = u‘int’

If the link is injectable, the previously mentioned methods can be applied, and the error values returned by the website can also distinguish the injection type. This is seen below.

def CheckError(html):

    
DbType = ‘’

    
if html.find(‘Microsoft OLE DB Provider for SQL Server’)! = -1:

        
DbType = u‘mssql’

….

    
elif html.find(‘Microsoft JET Database Engine’)! = -1 or html.find(‘[ODBC Microsoft Access Driver]’)! = -1 or html.find (‘[Controlador ODBC Microsoft Access]’)! = -1:

        
DbType = u‘access’

    
elif html.find(‘Microsoft OLE DB Provider for ODBC Drivers’)! = -1 and html.find(‘[MySQL]’)! = -1:

        
DbType = u‘mysql’

….

    
elif html.find(‘Microsoft’)! = -1 and html.find(‘line’)! = -1:

        
DbType = u ‘unknown’

….

    
return IsShowError, DbType

Different detection functions are named according to the different database types. Access, for example, must have a hidden data table, “msysaccessobjects”, in the database. Therefore, testing special data tables helps determine whether the site host uses an Access database.

def CheckAccess(self):

nRet = CheckKey(self.conf,"and 0<(select count(1) from msysaccessobjects)")

    
if nRet = = True:

        
self.conf.DbType = ‘access’

        
return True

    
else:

            
return False

After detection of the Access database, this system first uses “Union” to determine which bytes encompass the database’s content. If this instruction is supported, the database content can be displayed using blasting technology.

#### 3.3 Scheduler

The scheduler in this system serves two functions: the crawler’s crawling priority scheduling and the injection’s testing priority scheduling. The main purpose of priority scheduling is to detect more SQL injection threat links in less time, which improves the system detecting efficiency.

In order to generate an injection library, this study analyzed the link structure of the SQL threat cases that were collected from the Exploit Database [50] and WooYun [51]. Using the data collected by these two vast databases, SQL injection threat links are determined faster.

Every newly collected link is saved in the crawler queue and injection queue. The scheduler gives the new link different priorities, according to the relevance judgment between the new link structure and the library information. Injection testing results are returned to the crawler module, and the crawler queue adjusts the data priorities, which guides the crawler to select a prior website to crawl. The link with the threat of an SQL injection can quickly be detected using these priority adjustments.

The link structure is divided into three parts: domain, directory name, and parameters. The relevance among them has three levels. Links with the same directory names have the highest level of relevance. Links with different directory names but the same parameters have a medium level of relevance. Links with different directory names and different parameters have the lowest level of relevance, meaning the relevance degree between them is zero.

## Experiments

### 4. Real experimental analysis

In order to verify the system’s validity, we conducted two experiments.

#### 4.1 Experiment 1

This experiment tested 30 university websites, which were randomly sampled from the top 50 of the Quacquarelli Symonds 2013 university ranking list [40]. E-mail addresses were gathered first, and then the injectable URLs were determined. This analysis was done on a single computer running Windows 8, with an Intel Core I7 3.9 GHz (six-core processor) and 16 GB RAM. Each university website was allotted a maximum of 48 hours for analysis, although some analyses terminated before that time limit.

**Fig 6:**
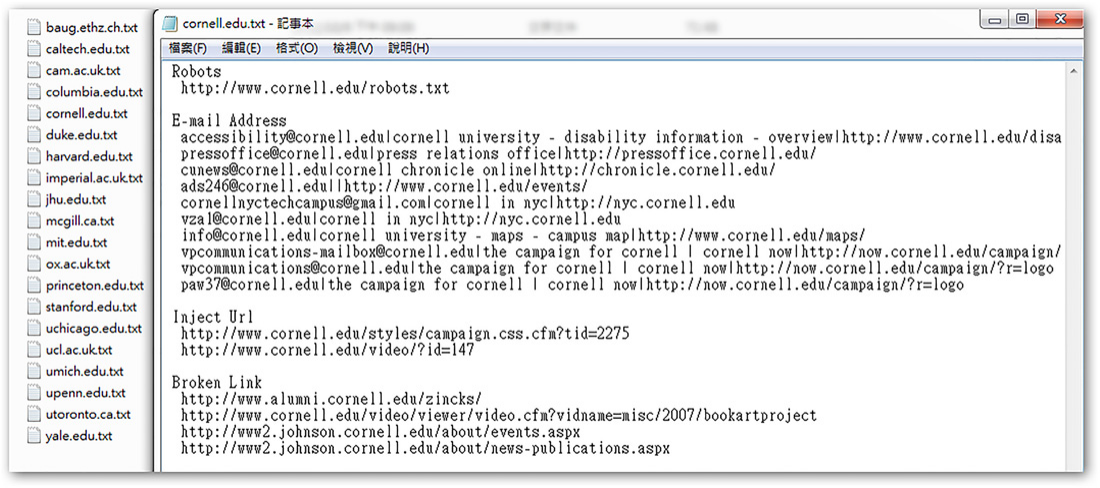
Program output, showing e-mail addresses, injectable URLs, and broken links.

Table 1 shows the number of e-mail addresses and injectable URLs found after 48 hours spent mining the 30 university websites. Six universities exposed over 10,000 e-mail addresses, and nine universities had URLs that could be injected. In total, 63,522 e-mail addresses and 82 injectable URLs were detected in this experiment. Fig. 6 shows an example of the system’s direct output in terms of e-mail addresses, injectable URLs, and broken links.

**Table 1:** University e-mail number and injectable URL statistics.

**School**	**Website**	**E-mail Number**	**Injectable URL Number**
Massachusetts Institute of Technology	web.mit.edu	5241	0
Harvard University	www.harvard.edu	38	0
University of Cambridge	www.cam.ac.uk	759	1
University College London	www.ucl.ac.uk	2389	6
Imperial College London	www.imperial.ac.uk	1	0
University of Oxford	www.ox.ac.uk	3524	2
Stanford University	www.stanford.edu	9611	8
Yale University	www.yale.edu	9621	2
University of Pennsylvania	www.upenn.edu	6785	1
Cornell University	www.cornell.edu	2212	3
University of Edinburgh	www.ed.ac.uk	6254	0
University of Toronto	www.utoronto.ca	1437	14
Ecole Polytechnique Fédérale de Lausanne	www.epfl.ch	1	0
McGill University	www.mcgill.ca	1236	9
University of Michigan	www.umich.edu	310	1
University of Hong Kong	www.hku.hk	712	12
Australian National University	www.anu.edu.au	394	0
Ecole normale supérieure, Paris	www.ens.fr/?lang=fr	353	0
Northwestern University	www.northwestern.edu	2765	0
University of Bristol	www.bristol.ac.uk	4718	11
The University of Melbourne	www.unimelb.edu.au	514	6
The University of Tokyo	www.u-tokyo.ac.jp/en/‎	8	0
The University of Manchester	www.manchester.ac.uk	646	1
The Hong Kong University of Science and Technology	www.hku.hk	612	1
Kyoto University	www.kyoto-u.ac.jp/en	31	0
Seoul National University	www.snu.ac.kr	4	0
University of Wisconsin-Madison	www.wisc.edu	43	0
The University of Sydney	www.sydney.edu.au	0	0
The Chinese University of Hong Kong	www.cuhk.edu.hk	3296	4
University of California, Los Angeles	www.ucla.edu	7	0

**Fig 7:**
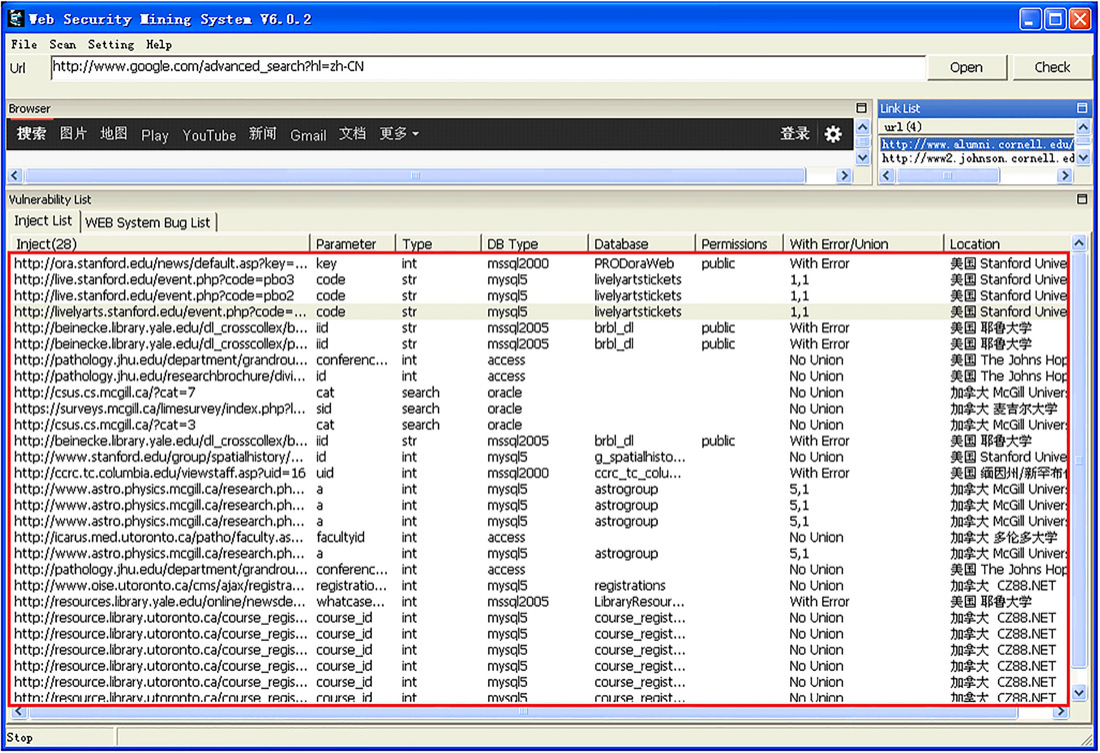
Detailed analysis of injected URLs.

**Fig 8:**
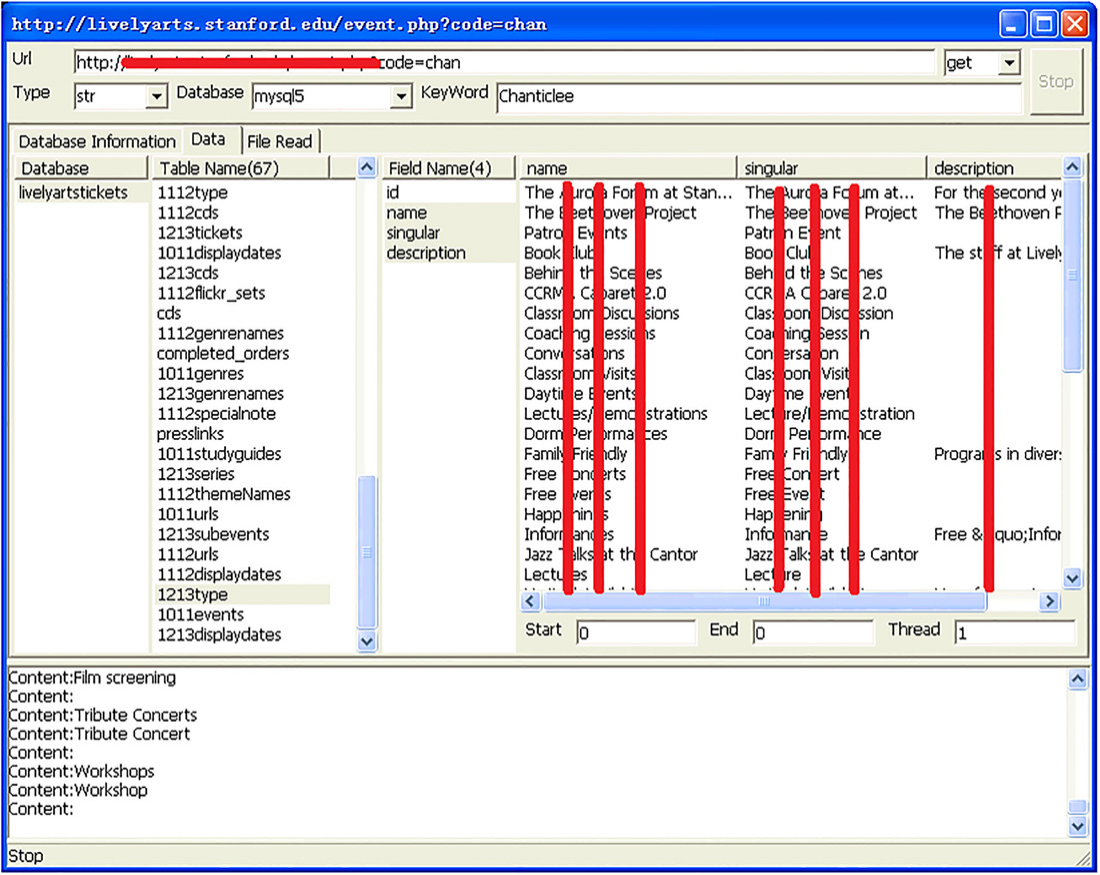
Database content revealed by an injection attack.

The data from Table 1 is further summarized in Fig. 7 and Fig. 8, which display the distributions of e-mail and injectable URL counts by university. According to these two figures, universities having over 1,000 leaked e-mail addresses account for 80% of the total number of universities. This experiment shows that most universities do not take extra steps in order to process the “@” symbol, such as changing “@” to “at” or replacing it with an “@” picture. The injectable URL inspection resulted in nine universities having injection vulnerabilities, which could let hackers gain access to the underlying databases and exploit the information for a variety of malicious purposes.

**Fig 9:**
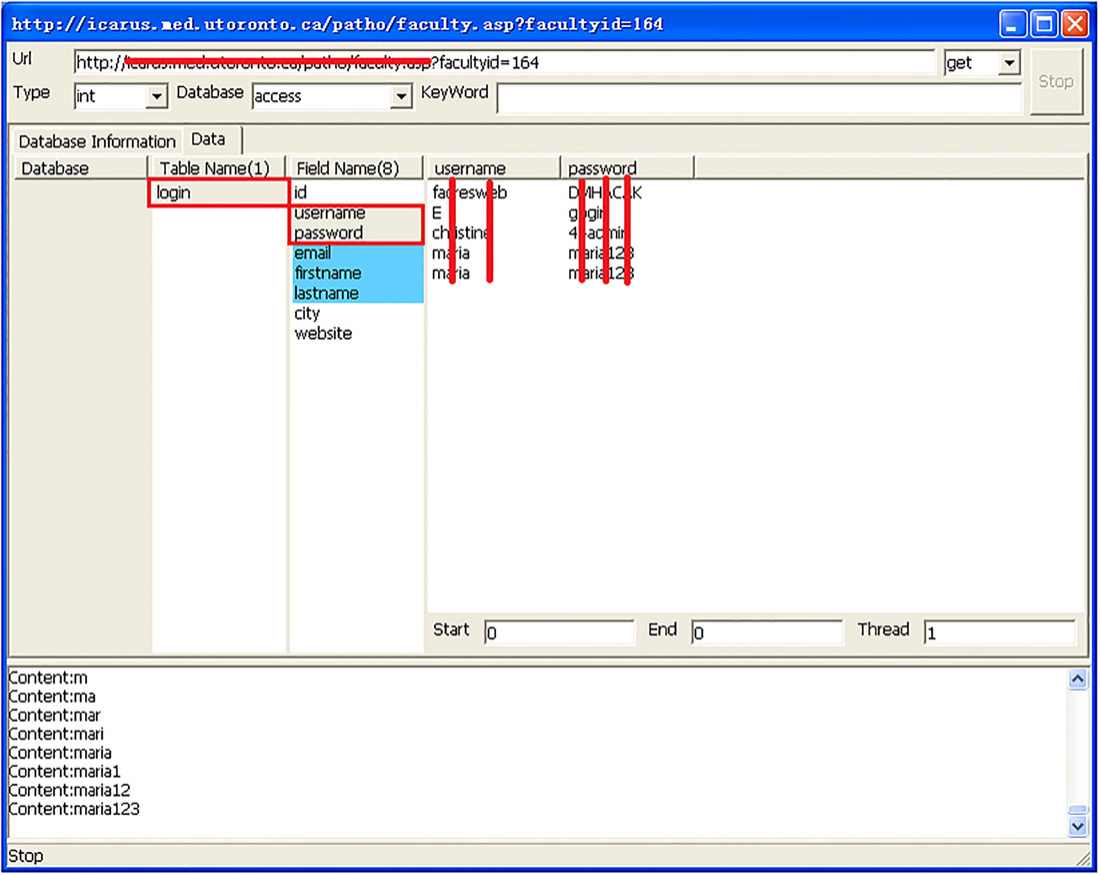
Accounts and passwords stored in the database.

Fig. 7 shows details of the 30 university websites, including database types, database names, and the specific formats used for injection attacks. Upon further exploration of the databases, we were able to identify database content, as is shown in Fig. 8. Additionally, we found some databases that stored user account passwords in clear text rather than hashing them. This can be seen in Fig. 9.

#### 4.2 Experiment 2

This experiment was done on a single computer running on Windows 7, with an Intel Core I7 3.4 GHz (four-core processor) and 8 GB RAM. Three department websites of the National Chung Cheng University (CCU) were targeted: the Department of Communications Engineering (COMM.CCU), the Department of Electrical Engineering (EE.CCU), and the Department of Computer Science and Information Engineering (CSIE.CCU).

**Fig 10:**
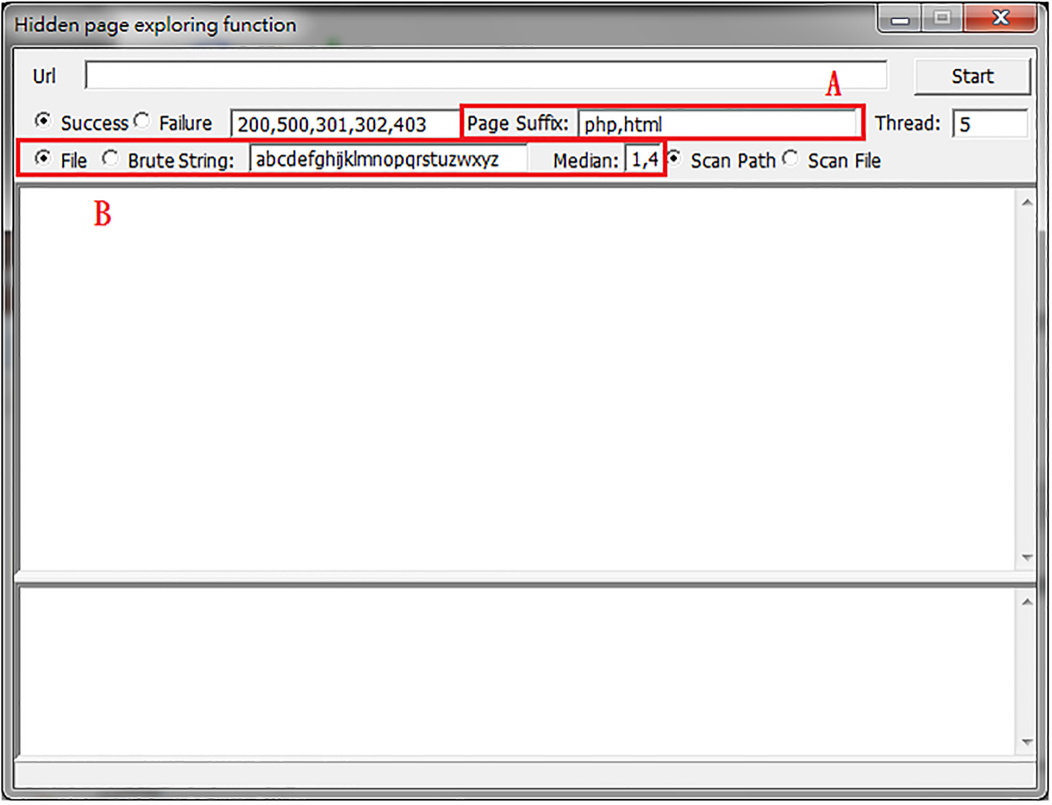
Interface for hidden page exploring function.

Typically, the remote managers’ pages are hidden in locations without explicit link URLs, which makes it difficult for outsiders to decipher. These pages often return useful information for detection, and sometimes they reveal key parameters of the website database. The WIDAS hidden page exploring function is shown in Fig. 10. Area A is added to the interface in order to enter a web language because there is an ever-increasing amount of web languages, such as HTML, ASP, PHP, CGI, and JSP, being used. In area B, when searching for hidden pages, the page name defined by the syntax dictionary is selected for scanning or exhaustive searching. Exhaustive searching examines the given characters and length one at a time. This means it takes longer, but it also has wider coverage. Page names defined in the syntax dictionary can be added or deleted according to the currently predominant naming rules.

This experiment ran the WIDAS hidden page exploring function in three department web pages and discovered the five recessive results listed in Table 2.

**Table 2:** Statistical results of WIDAS hidden page exploring function.

List of HTTP Status Codes	Contents
200 OK	Standard response for successful HTTP requests. The actual response will depend on the request method used. In a GET request, the response will contain an entity corresponding to the requested resource. In a POST request the response will contain an entity describing or containing the result of the action.
301 Moved Permanently	This and all future requests should be directed to the given URI.
302 Found	This is an example of industry practice contradicting the standard. The HTTP/1.0 specification required the client to perform a temporary redirect (the original describing phrase was “Moved Temporarily”), but popular browsers implemented 302 with the functionality of a 303 “See Other.” Therefore, HTTP/1.1 added status codes 303 and 307 to distinguish between the two behaviors. However, some web applications and frameworks use the 302 status code as if it were the 303.
403 Forbidden	The request was a valid request, but the server is refusing to respond to it. Unlike a 401 Unauthorized response, authenticating it will make no difference.
500 Internal Server Error	A generic error message, given when an unexpected condition was encountered and no more specific message is suitable.

In the three department websites, WIDAS obtained two types of e-mail addresses: dominant and recessive. Dominant e-mail addresses are usually open to the public, belong to teachers or administrators, and are located in the upper part of the web page. Recessive e-mail addresses are usually in deep web domains, such as old discussion boards, old workshops, or various subject pages. The experimental results are shown in Table 3.

**Table 3:** E-mail addresses in three department websites.

	**COMM.CCU**	**EE.CCU**	**CSIE.CCU**
**Dominant e-mail amount**	82	51	114
**Dominant URL**	http://www.comm.ccu.edu.tw	http://www.ee.ccu.edu.tw	http://www.cs.ccu.edu.tw
**Recessive e-mail amount**	31 (as shown in Fig. 11)	10 (as shown in Fig. 12)	53 (as shown in Fig. 13)
**Recessive URL amount**	16 (as shown in Fig. 14)	2 (as shown in Fig. 15)	8 (as shown in Fig. 16)

**Fig 11:**
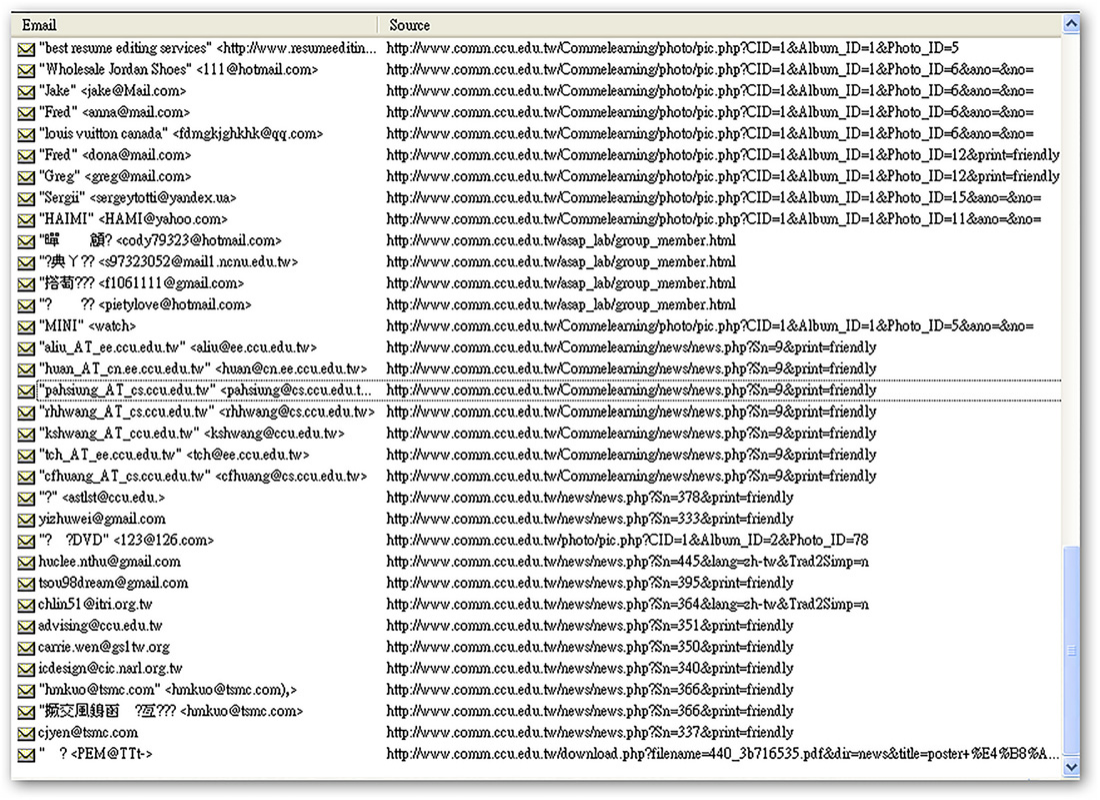
Recessive e-mail amount from COMM.CCU website.

**Fig 12:**
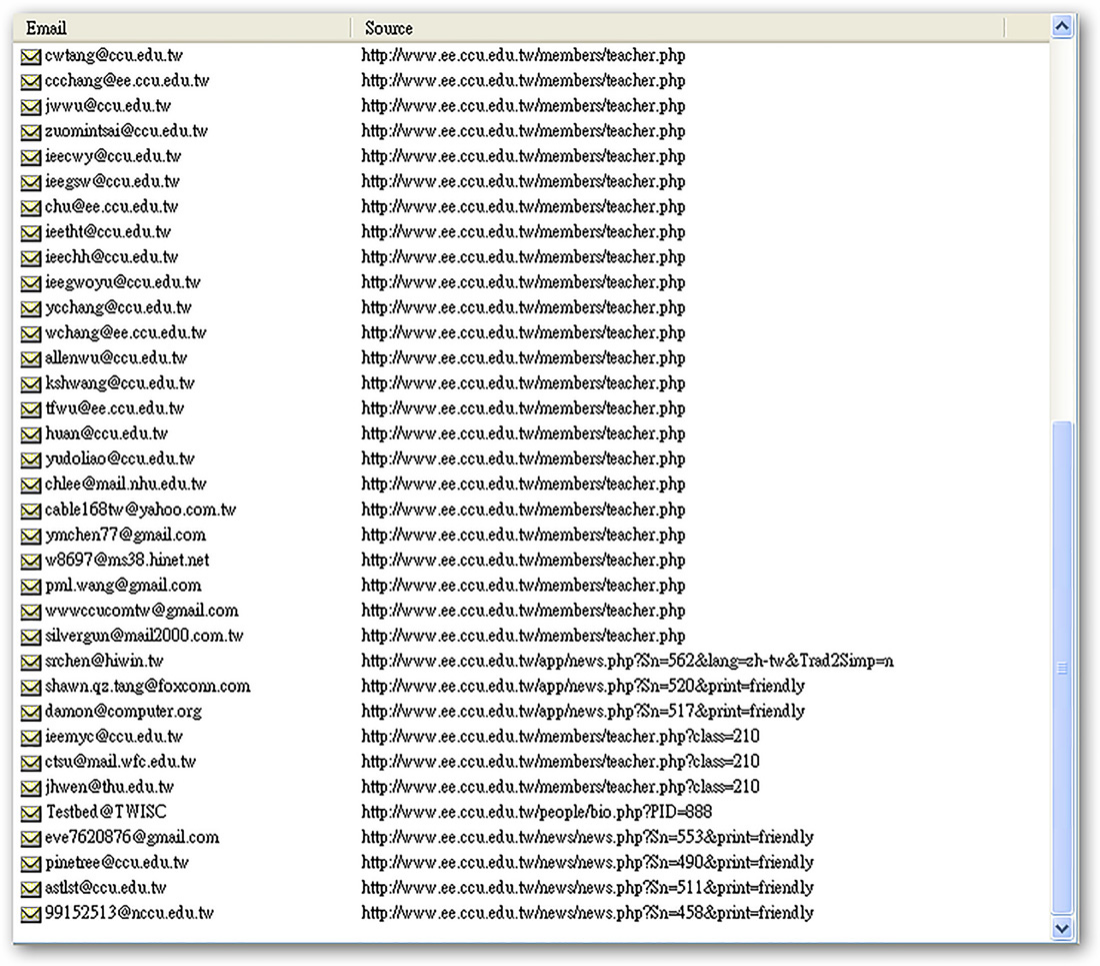
Recessive e-mail amount from EE.CCU website.

**Fig 13:**
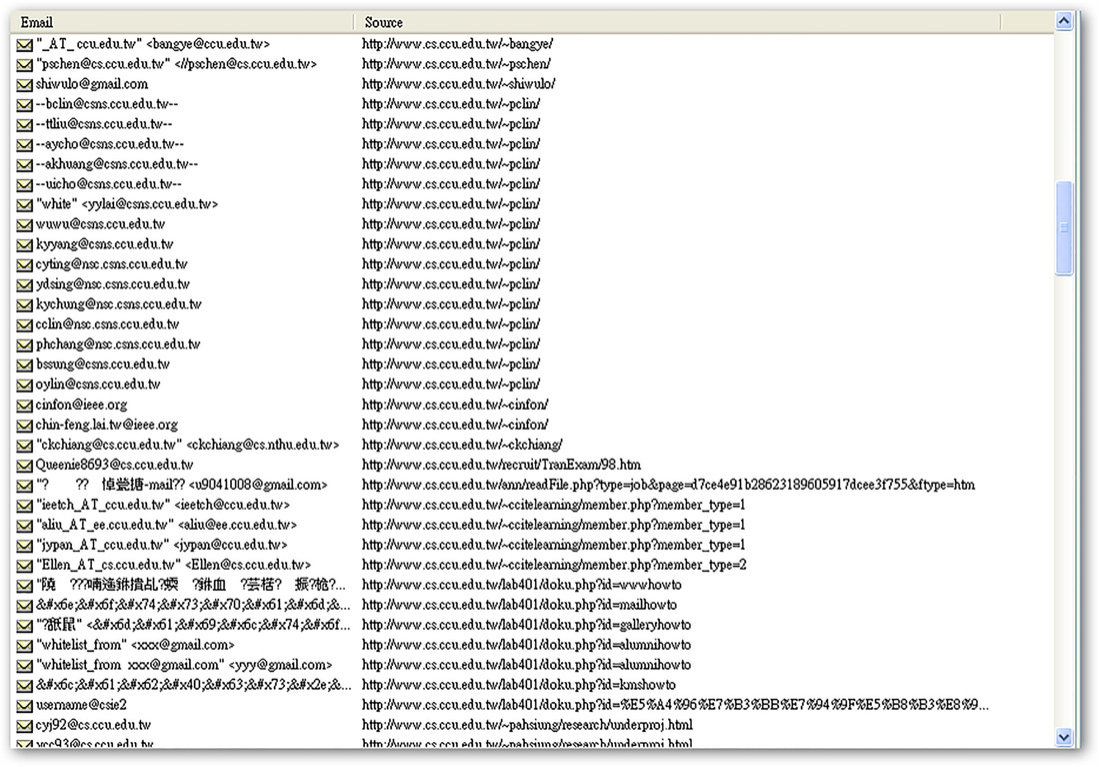
Recessive e-mail amount from CSIE.CCU website.

**Fig 14:**
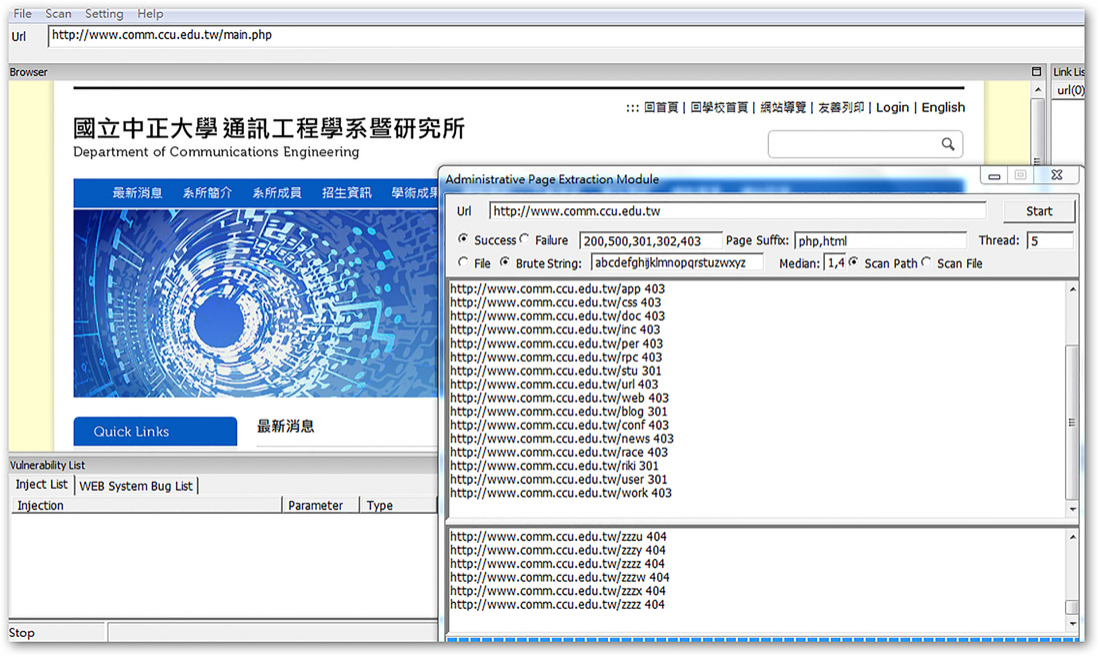
Recessive URL amount from COMM.CCU website.

**Fig 15:**
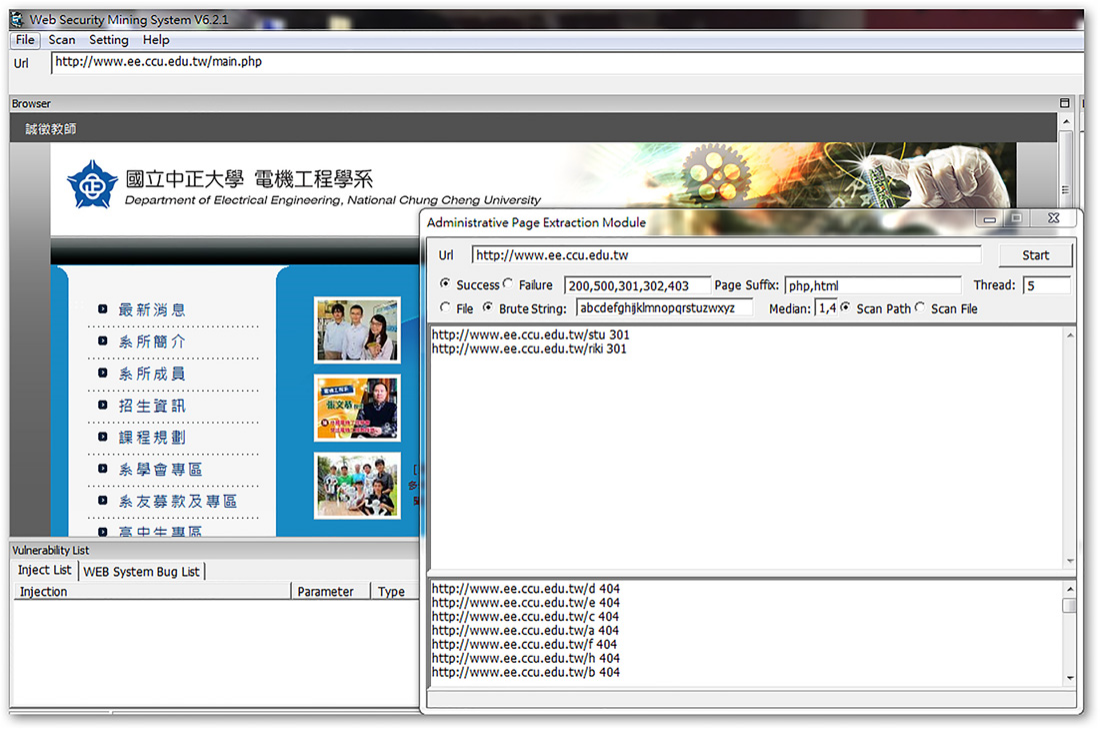
Recessive URL amount from EE.CCU website.

**Fig 16:**
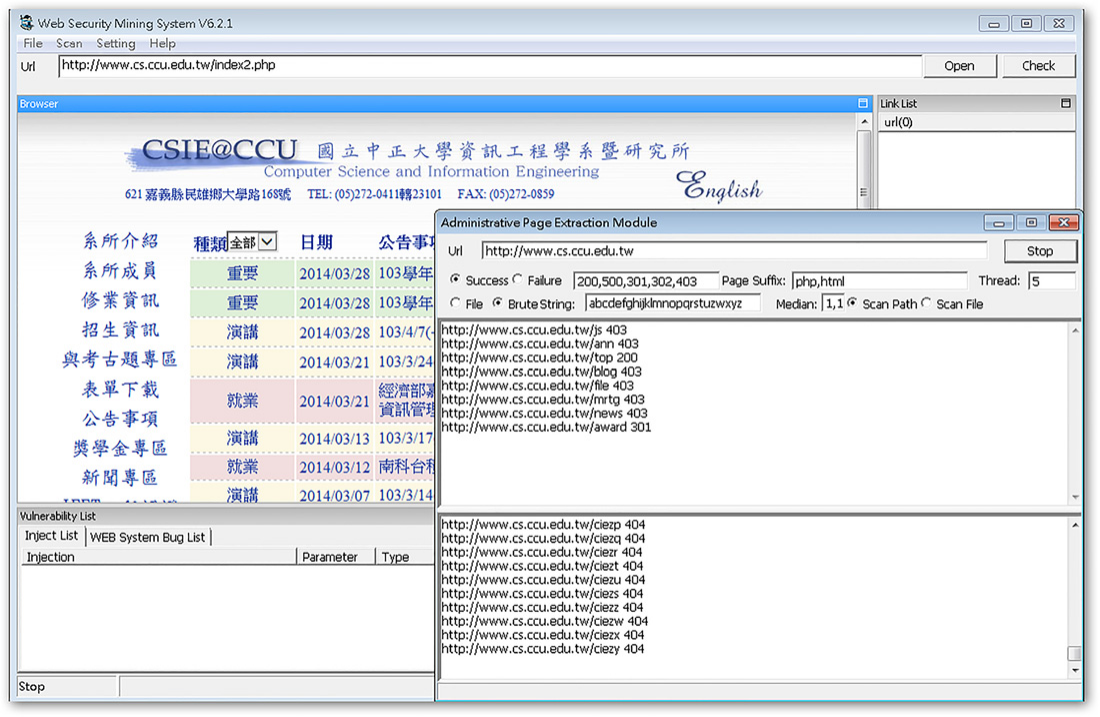
Recessive URL amount from CSIE.CCU website.

Recessive email amounts for each department website listed in Table 3 are shown in Figs. 11–16.

## Results

### 5. Comparison with different applications

There are various applications used for testing security vulnerabilities, such as the Acunetix Web Vulnerability Scanner, aidSQL, Gamja, and Grabber. They are based on PHP, ASP, ASP.NET, VB.NET, C#, Java, or some other programming language. The main performance comparisons with well-known applications are listed in Table 4. The numeric representations are as follows: “1” builds crawlers and explores the entire site’s architecture; “2” searches and stores the entire web page’s links; “3” mirrors the entire website; “4” outputs a statistical analysis report; “5” searches for the web address according to keywords; “6” crawls all e-mail accounts on the website; “7” scans web weakness using keywords and help from search engines; “8” deeply mines weaknesses in a single site; “9” has a mobile version; “10” analyzes web syntax; “11” detects and analyzes hidden web pages; “12” downloads different types of files on the website; “13” tests weak passwords; “14” scans live IPs within a specified network domain and analyzes its properties; “15” updates website vulnerabilities; and “16” runs complex pages such as Ajax.

**Table 4:** Performance comparison of vulnerability mining software.

Software / Function	1	2	3	4	5	6	7	8	9	10	11	12	13	14	15	16
Teleport Pro	√	√	√		√							√				
Black Widow	√		√													
Win Web Crawler	√	√			√											
Visual Web Spider	√	√			√											
JOC Web Spider	√		√									√				
Gyxi’s Image Spider	√											√				
DRK Spider	√															
HDSI2005	√						√	√								
Wget	√		√													
HTTrack Web Copier	√		√													
Acunetix WVS	√	√		√			√	√		√						√
NTOSpider	√	√		√			√	√		√						
Netsparker	√	√		√			√	√		√						√
IBM AppScan	√	√		√			√	√	√	√	√					√
HP WebInspect	√	√		√			√	√		√	√					√
Syhunt Dynamic	√	√		√			√	√		√						
Burp Suite	√	√					√	√		√	√		√			√
N-Stalker Enterprise	√	√		√			√	√		√						√
WebCruiser	√	√		√			√	√	√	√						
Zed Attack Proxy	√	√		√			√	√		√			√			
IronWASP	√	√		√			√	√		√						
N-Stalker	√	√		√			√	√		√						
WebSecurify	√	√		√			√	√		√						
WIDAS	√	√	√		√	√	√	√		√	√	√	√	√	√	

As seen in Table 4, the proposed system has obvious advantages and comprehensive functions, especially as it takes into account the entire website mirror, the web address search using keywords, the situation in which all e-mail accounts crawl in the website, the hidden web page detection and analysis, different types of files on the website download, and website vulnerability updates.

### 6. Response strategies

E-mail address leakage and web database leakage are currently the two most serious information vulnerabilities. In contrast to traditional phishing attacks, bouncer list phishing [41–43], a new phishing attack, can lock on specific targets, and only these targets can visit the phishing website. Therefore, this attack pattern avoids detection or, at least, delays the detection time. Here, the attacker sends e-mails and attaches malicious links. When the user clicks on the link, the attacker will first verify his or her identity and then load the phishing web page if the user is on the target list or send a message, such as “can’t find the page”, if not. This is similar to a VIP party, in which only the guests on the invitation list can attend. This new phishing is also a type of APT attack. It is strategic, it is not a single event, and it usually lasts for a long time. Therefore, protection methods must be strong, and users should form good information operation habits.

#### 6.1 Characteristics of APT attacks

1. *Locked particular targets*: A planned, organized, information-stealing attack against specific governments or companies may take a few days, weeks, months, or years.
2. *Fake letters*: By sending malicious social engineering e-mails to locked targets, the attacker first obtains an opportunity to install malicious applications on the target computer.
3. *Low and slow operation*: In order to continually steal an administrator’s account and password without being detected, the malware must always be self-hidden.
4. *Customized malicious components*: Aside from ready-made malware, attackers also use malicious customized components.
5. *Remote control tools*: A remote-control architecture similar to botnets can be created to regularly copy files with potential value, send them to the attack command, and control the server.
6. *Information Delivery*: Filtered sensitive confidential data may be encrypted and sent outside by malwares.

#### 6.2 E-mail protection methods

Given these features, we find that hackers quickly determine attributes of e-mail owners from public websites. Therefore, e-mails on public websites need to be protected from web crawlers while the access of legitimate viewers is not affected. This paper proposes the following methods:

(1) *Replace “@” and “*.*” in e-mail addresses by other symbols*, *such as* “abcdefg(at)hotmail(dot)com” *or* “admin[at]mail[dot]com.”
(2) *Change the code direction using CSS “unicode-bidi” or “direction”*.

<style>

span.codedirection {unicode-bidi:bidi-override; direction: rtl;}

</style>

<p><span class = “codedirection”> moc.liam@nimda </span> </p>

(3) *Utilize the CSS “display*:*none”*.

<style>

p span.displaynone {display:none;}

</style>

<p>admin@<span class = “displaynone” > null</span> mail.com </p>

(4) *Encrypt using ROT13*.

<script>

document.write(“<nuers = “znvygb:fvyinasbbone10@ gvyyyngr.pbz” ery = “absbyybj”>”.replace(/[a-zA-Z]/g, function (c) {return String.fromCharCode((c< = “Z”?90:122)> = (cc = c.charCodeAt (0)+13)? c:c-26);}));

</script>admin’s Mail</a>

(5) *Replace JavaScript code (I)*.

<script>

function TagReplace(str)

{

    
str = str.replace(“Cople’s”,“admin”);

    
str = str.replace(“Mail”,“/mail.com”);

    
str = str.replace(“/”,“@”);

    
return(str)

}

document.write(TagReplace(“admin’s Mail”))

</script>

(6) *Replace JavaScript code (II)*.

<script>

var name = “your admin@mail.com account name”;

var domain = “your admin@mail.com sever”;

document.write("<a href = “mailto:”+name+“@"+domain+"">");

document.write(name+"@"+domain+"</a>");

</script>

(7) *Encrypt using JavaScript code*. This method ensures that robots are unable to get the real address.

<script>

function hivelogic_enkoder(){var kode =

”kode = “oked” = rnhg%@nrgh%_n@gr_h_%n_g@_rh___

%__u{_k@zj}ioskt(4gxnzk.&B__"+"Cx(lgbrsuoizv@

kuirF4wtiws4(uzbz&kobrbCD(5(DB /g____(A%___>{@

**>iru+l@3>l?nr"+"gh1ohqjwk>l.,~f@

nrgh1fkduFrghDw+l,06>li+f?3,f.@45;>{.@

Vwulqj1iurpFkduFrgh"+"+f000r,hn{g_@>__@*%i{u*l >

3rl++ @r>h?onqgw10h,jlk@4,>{.@5r~h.fndgD1+k.u,"+

"wnlg41.kruhwfldD00+0,rnhg{@+.?lrnhgo1qhwjBkrnhgf1dkDu+

wrnhgo1qhwj0k,4*"+" =, *>_>@%*{i*u>lr3+l@+

>r?hnogq1wh0j,kl4@>,.{5@~r.hnfgd1Dk+u.w,ln4g.1rkhufwd"+

"lD+00,0nrgh@{.+l?nrgh1ohqjwkBnrgh1fkduDw+

nrgh1ohqjwk04, = **,%>{>*@>*ri+"+"u@l>3?ln+

gr1hhojqkw40>,.l5@~,.{n@gr1hkfudwDl+4.,rnhgf1dkDu+

w,l000rnhg"+"{@+.?lrnhgo1qhwjBkrnhgf1dkDu+

wrnhgo1qhwj0k,4* =, *">x;’ =; ’of(r = i;0<iokedl"+

".netg;h+i)+c{k = do.ehcraoCedtAi(-);3fic(0<c) = +21;8+xS

= rtni.grfmohCraoCedc(}"+")okedx = “;x = ’’;

for(i = 0;i<(kode.length-1);i+ = 2){x+ = kode.charAt(i+1)+

kode.ch"+"arAt(i)}kode = x+(i<kode.length?kode.charAt

(kode.length-1):’’);";var i,c,x;while(eval(kode));}

hivelogic_enkoder();

</script>

(8) *Hide e-mails*. The following codes can hide e-mails for a few seconds. Although difficult for robots to collect, it is easy for normal users to directly read.

<script>

var BtxVIdHlXs = “qPaqzK”;var LURRQZ =

"@WlA.com";var qEFIxznKla =

"LbDmvBaAm";var pGJqYOsAsB =

"@kfgTpD.com";var nlEeReX = “cople.cn”;var HASfTupp =

"@qq.com";var PDPtUCBXl = “FIHQDolF”;var YjmaNjK =

"@WUT.com";var VxmEjRWCF = 2005;

setTimeout("dOSQjupmqhAVBEJ()",VxmEjRWCF);

function dOSQjupmqhAVBEJ()

{document.getElementById("YVeorjwmX").innerHTML =

nlEeReX + HASfTupp;}

</script>

<span id = “YVeorjwmX”>admin@mail.com Loading…</span>

(9) *Utilize CSS pseudo-classes*. Insert “::before” and “::after” into e-mail usernames and domain names to the right of the “@” symbol. Web spiders usually cannot see CSS, but they can see the “@” symbol. The following example hides “john@gmail.com”.

<style>

  
my-admin@mail.com::after {

    
content: attr(data-domain);

  
}

  
my-admin@mail.com::before {

    
content: attr(data-user);

  
}

</style>

<!—set user name and realm name of admin@mail.com through data-user and data-domain—>

<my-admin@mail.com data-user = “john” data-domain = “gmail.com”>@ </my-admin@mail.com>

(10) *Use JavaScript’s “onclick” event*. E-mail addresses can be outputted as mailto links by replacing the characters “.” and “@” with text. Adding the “onclick” event converts these replacements.

<a href = “mailto:adminATmailDOTcom”

   
onclick = “this.href = this.href

              
.replace(/AT/,‘@’)

              
.replace(/DOT/,‘.’)”

>connect me</a>

(11) *Disorder arrays*. Divide an e-mail address array into several parts, output the correct order using JavaScript, and add it to the web page utilizing the “.innerHTML” attribute.

<span id = “admin@mail.com”></span>

<script>

  
var parts = [“john”, “abc”, “com”, “.”, “@”];

  
var admin@mail.com = parts[0] + parts[4] + parts[1] +

 
parts[3] + parts[2];

  
document.getElementById("admin@mail.com").innerHTML =

admin@mail.com;

</script>

(12) *Utilize the Google reCAPTCHA Mailhide tool*. This tool helps protect the receiver box by requesting users to view the e-mail address only after correctly verifying the reCAPTCHA questions. This mechanism prevents e-mail addresses from being automatically found by spammers.
(13) *Implement “antispambot” in WordPress*. This function transforms e-mail addresses into ones that cannot be identified by robots but can be displayed by browsers. The following codes are added in functions.php theme files.

add_shortcode (‘admin@mail.com’, ‘wpjam_admin@mail.com_shortcode_handler’);

function wpjam_admin@mail.com_shortcode_handler ($atts, $content = ’’) {

    
extract (shortcode_atts (array(

        
‘mailto’ = > ′0’

    
), $atts));

return antispambot ($content, $mailto);

}

(14) *Employ the AntiSpamBot Shortcode plugin tool*. This tool is easy to master. After uploading and activating, enter the e-mail address, you-e-mail-address@e-mail.com. The source code of the e-mail address is given by:

you-email-address@ email.com

#### 6.3 Database protection methods

During the penetration-testing phase, we found many website databases with flawless, solid code management. We then summarized these practical approaches that prevent SQL injection.

1. *Clearly define users’ rights when accessing a database*. If a normal user embeds a DROP TABLE statement in the SQL query syntax, the program must decide whether or not to execute it. The Drop grammar is relative to the basic database object, so legitimate users must have the corresponding permissions. Unless necessary, terminal users, i.e. the application system operators, do not need the right to establish or delete database objects. Even if the SQL statement has been implanted with malicious operation grammar or program code, the action will not be executed because the rigorous access control is restricted to the user operation. Therefore, it is better to distinguish system administrator users from ordinary users in the access architectural design. This greatly reduces the harm caused by SQL injection attacks.
2. *Use parameterized query syntax*. When writing SQL query syntax, if a user’s input variables do not have a direct, dynamic connection to the SQL query syntax and are passed as parameters, data hidden codes SQL injection attacks can be effectively avoided. In other words, the user’s inputs cannot directly be incorporated into the SQL query syntax. To avoid attack cases, the user’s inputs must be filtered, or parameterized queries must be employed to deliver the user’s input variables. Adopting these measures ends most data hidden codes SQL injection attacks. Unfortunately, few database systems support parameterized statements, and developers should use this method when designing a system.
3. *Check and verify user input data*. Many corresponding ready-to-use tools exist that check and verify user input data. In the SQL server database, there are several user input validation tools that can be used by administrators to deal with SQL injection. For example, if only the required value is accepted and content containing binary data and comment characters is not filtered and validated, then improper attack grammar will not be implanted and some buffer overflow attacks, as well as other related attack techniques, can be prevented. Testing the data type, length, format, and range in order to validate user input data is one of the most common and effective precautions against data hidden codes SQL injection attacks.
4. *Use the built-in security parameters of the SQL server database*. In order to reduce the negative influence of data hidden codes SQL injection attacks, Microsoft specially designed some relatively safe SQL parameters for managers in the SQL server database. In the database design process, developers should use these parameters to prevent malicious SQL injection.
5. *Effectively prevent data hidden codes SQL injection attacks in the N-Tier architecture*. Many kinds of Internet applications currently adopt a 3-Tier or N-Tier application system architecture. In multiple application architectures, the user should be allowed to enter the data area only after verification, and attention must be paid to each tier. Both the client and database interfaces should adopt corresponding measures in order to prevent data hidden codes SQL injection.
6. *Use professional code vulnerability scanning tools to find the implied leakage for application systems*. Under the assistance of professional vulnerability scanning program code analysis tools, such as white box, application system developers can quickly and effectively find all possible attack code areas. Database administrators and application system developers should take active measures to prevent SQL data hidden codes attack in order to ensure attackers do not know how to start attacks.

## Conclusions

In our study, code review aided the static analysis [44], and penetration testing assisted the dynamic analysis [52]. The testing results of our static and dynamic analyses have limits. In order to improve network application security, penetration testing is indispensable, and security maintenance work is more successful when penetration testing is regularly undertaken.

Various automated programs that are used to collect data exist in the Internet environment at any given moment. During the experiments, we discovered that many academic websites do not specially treat or cloak the characteristics of “@” in e-mail addresses or of robots.txt files. The proposed system easily collected multiple e-mail addresses. Malicious users can automatically send dangerous Trojan virus e-mails to these addresses and cause security problems.

Traditional technologies, such as firewall access control, intrusion prevention systems (IPSs), and e-mail security gateways (ESGs), cannot meet the demands of current and future information defense. An APT is an advanced, continuous, and target-type attack. As opposed to traditional spammers, APTs adopt a long-term targeted penetration. In order to prevent attacks from spam and APTs, protecting public e-mail accounts on websites becomes of great importance.

In addition, this research also found that many databases stored passwords in clear text style, which may be easily utilized by hackers to impersonate permissions when they obtain the database contents. Software applications are a good starting point, but they will not fend off attacks from those who want to break software protections and steal useful information. Therefore, our results remind us that the encryption of data storage is as important as website design and database management.
